# Supplementary material for: Immune infiltration and prognosis in gastric cancer: role of NAD+ metabolism-related markers
Source: PeerJ. 2024 Jul 31;12:e17833. doi: 10.7717/peerj.17833 (PMC11297443; doi:10.7717/peerj.17833)
Supplement: Supplemental Information 12 [file peerj-12-17833-s012.docx]

1.2.7.1 RNA extraction steps: (All items used are treated with RNA-removing enzyme to prevent the degradation of extracted RNA)

1) The tissues stored in the -80 ° C refrigerator were taken out, weighing about 100mg, mashed in the grinder and then added 900μL of Trizol reagent. After the tissues were fully ground to completely lysate the cells, the tissues were moved to 1.5mL EP tube with RNase removed and left for 10 min at room temperature.

2) Add 300μL chloroform to each centrifuge tube, shake the centrifuge tube with a scroll machine for 30 sec, stand on the ice box for 5min, and then centrifuge at 13000rpm for 15 min at 4 ℃.

3) After centrifugation, the solution is divided into three layers, and the supernatant is taken and transferred into a 1.5mL centrifuge tube (in an ice box or on the ice).

4) Add isopropyl alcohol in the same volume as the supernatant, mix it upside-down, stand for 15 min (in an ice box or on ice), and centrifuge at 13000rpm for 10 min at 4 ℃.

5) Remove the supernatant, wash and precipitate 1 mL anhydrous ethanol solution per tube, and then centrifuge at 7200rpm for 5 min at 4 ℃.

6) Remove the ethanol solution and let it dry and precipitate for 5-10 min.

7) Add 24 μL DEPC water to dissolve RNA (can be frozen for a long time at -20℃).

1.2.7.2 Detection of RNA quality:

To ensure the quality of RNA, RNA electrophoresis was used. A 1% agarose gel was prepared, 4µLRNA stock solution and 1µL 6× nucleic acid sample loading buffer were thoroughly mixed, and the sample was loaded, 180V electrophoresis was performed for 15 minutes. After electrophoresis, the gel was observed and photographed in the gel imaging system. From top to bottom, there are three bands of 28S, 18S and 5S. If there is a tail, it indicates RNA degradation; if there is a bright band above 28S, it indicates DNA contamination. Generally, if the 28S and 18S bands are bright and clear, and the brightness of 28S is more than twice that of the 18S band, the RNA is considered to be of good quality.

1.2.7.3 Detection of RNA concentration:

According to the absorption values at 260 nm and 280 nm measured by ultraviolet spectrophotometer, the RNA concentration was calculated according to the following formula: RNA stock solution concentration = OD260 × dilution factor × 40 (ng/μL).

1.2.7.4 cDNA operation steps: Reverse the kit instructions according to Takara

1) The following reagents were successively added into the EP tube to prepare sample A (10µL in total).

primer 1µL

dNTP Mixture(10mM each) 1µL

Template RNA 2µL

RNase free dH2O 6µL

2) Put the reagent A in the EP tube into the 65℃ water bath for 5min, and then immediately put it into the ice.

1.2.7.5 RT-qPCR Procedure:

1) Reaction system (20µL system) :

SYBR Green Mix 9µL

RT Product 2µL

Bulge-Loop miRNA Forward Primer (5µM) 0.8µL

Bulge-Loop miRNA Reverse Primer (5µM) 0.8µL

RNase-free dH2O to 20µL

2) PCR reaction procedure: 94℃ 30sec predenaturation 1 cycle; Denatured at 94℃ at 10sec, annealed at 65℃ at 15sec, extended at 72℃ at 10sec, and amplified for 40 cycles. When Real-time Q-PCR was used to detect RNA, different RNA samples had different reverse transcriptional efficiency, and U6 was used as the internal reference for correction of quantitative analysis of PCR products. △Ct is the Ct value of the target gene minus the Ct value of the reference gene U6, △△Ct= the experimental group △ ct-control group △Ct, and the expression ratio of mRNA of the experimental group relative to the control group is expressed by 2-△△Ct.
